# Associations between Pharmacological Treatment Patterns during the Initial Treatment Period and the Relapse or Recurrence of Anxiety Disorders: A Nationwide Retrospective Cohort Study

**DOI:** 10.3390/life13051197

**Published:** 2023-05-17

**Authors:** Junhyung Kim, Changsu Han, Moon-Soo Lee, Hyun-Ghang Jeong, Jae-Jin Kim, Seung-Hyun Kim

**Affiliations:** 1 Department of Psychiatry, Korea University Guro Hospital, Korea University College of Medicine, Seoul 08308, Republic of Korea; jhcabilover@gmail.com (J.K.);; 2 Department of Life Sciences, Korea University, Seoul 02841, Republic of Korea; 3 Department of Psychiatry, Yonsei University College of Medicine, Seoul 03722, Republic of Korea

**Keywords:** anxiety disorder, pharmacological treatment, relapse, recurrence, retrospective cohort study, Health Insurance Review and Assessment Service Data

## Abstract

Although the importance of proper pharmacological treatment for preventing the relapse/recurrence of anxiety disorders is well known, a real-world data-based study has not been conducted. We aimed to investigate the effect of the initial pharmacological patterns related to continuous treatment and the choice of medication on the relapse/recurrence of anxiety disorders. We used claim data from the Health Insurance Review and Assessment Service, South Korea, of 34,378 adults who received psychiatric medications, including antidepressants, after being newly diagnosed with anxiety disorders. We compared the relapse/recurrence rate in the patients receiving continuous pharmacological treatment with those who discontinued treatment early using Cox’s proportional-hazards model. Patients receiving continuous pharmacological treatment experienced a higher risk of relapse/recurrence than those who discontinued treatment. Using three or more antidepressants during the initial treatment period decreased the risk of relapse/recurrence (adjusted hazard ratio (aHR) = 0.229 (0.204–0.256)); however, the combined use of antidepressants from the beginning of treatment increased the risk (aHR = 1.215 (1.131–1.305)). Factors other than continuous pharmacological treatment should be considered to effectively prevent the relapse/recurrence of anxiety disorders. The active use of antidepressants, including switching or adding medications based on progress and frequent follow-up visits during the acute phase, were significantly associated with a reduction in the relapse/recurrence of anxiety disorders.

## 1. Introduction

Anxiety disorders are mental diseases accompanied by anxiety that affect an individual’s life [1]. Anxiety disorders are prevalent [2] and cause socioeconomic losses [3]. A high relapse/recurrence rate of anxiety disorders, with various lengths of follow-up, has been consistently reported [4,5]; a recent longitudinal study reported that only 51% of patients with an anxiety disorder and without a comorbidity had consistently recovered at a 2-year follow-up, and this number was only 26% at a 9-year follow-up [6]. Therefore, minimizing recurrence is essential to alleviating the social burden of anxiety disorders.

Pharmacological treatment is essential for treating anxiety disorders [7]. Although cognitive behavioral therapy (CBT) also plays an important role in treatment, most patients are initially treated with pharmacological agents owing to the drawbacks associated with CBT [7,8]. Patients with anxiety disorders typically begin their treatment in primary care [9], where CBT is often difficult to implement owing to the limitations of many settings [10]. Moreover, patients with anxiety disorders are less likely to be hospitalized due to their longer treatment duration and relatively low severity [11]. Therefore, establishing an appropriate pharmacological treatment strategy in an actual outpatient clinical practice is important.

International guidelines recommend continuous pharmacological treatment for 6–24 months to avoid the recurrence of anxiety disorders after the acute treatment phase [12]. The recommendation to continue pharmacological treatment is based on randomized controlled trial (RCT) discontinuation studies, which have reported higher recurrence rates among responders discontinuing pharmacological treatment than in the treatment continuation group [13]. Thus, the long-term use of pharmacological agents (especially antidepressants) has increased, with approximately two-thirds of those in the U.S. continuing antidepressants for at least 2 years [14]. However, most RCT discontinuation studies focused on recurrence only in patients with remitted anxiety disorders and examined recurrence only during the pharmacological treatment period; thus, the information on long-term prognosis after treatment is limited [15,16]. Moreover, as most RCT discontinuation studies examined the efficacy of the discontinuation of a single medication only, concluding whether continuous pharmacological treatment is an effective treatment strategy to prevent recurrence in actual clinical practice is impossible. Thus, to avoid unnecessary long-term pharmacological treatment, determining whether its continuous application is effective in preventing the recurrence of anxiety disorders using real-world data is essential.

Although a nationwide retrospective cohort study, using claim data, can provide meaningful information on the effects of interventions on recurrence [17], such studies have not yet been conducted. Additionally, international guidelines also recommend initially prescribing antidepressants, including selective serotonin reuptake inhibitors (SSRIs), serotonin–noradrenaline reuptake inhibitors (SNRIs), and tricyclic antidepressants, and appropriately substituting medication according to treatment progress [18]. However, whether the category of antidepressant or the number of antidepressant categories used during the treatment period impacts recurrence is unknown. Clinical information concerning the recurrence of anxiety disorders and pharmacological treatment can be obtained from the Health Insurance Review and Assessment Service (HIRA), which contains claim data from the entire South Korean population [17].

In this study, we aimed to investigate the associations of the initial pharmacological patterns with the recurrence of anxiety disorders in outpatient clinical settings. Taking advantage of a large, population-based sample, we investigated whether continuous or early discontinued pharmacological treatment was associated with the recurrence of anxiety disorders.

## 2. Materials and Methods

### 2.1. Study Participants

Diagnostic codes and medical prescriptions for each participant were obtained from HIRA. Participants were included if (a) their primary diagnosis was an anxiety disorder (International Classification of Disease, 10th version (ICD-10) code: F41.x) or a phobic anxiety disorder (F40.x) at 18–85 years old between 1 January 2013 and 31 December 2014 (the index period), and (b) their primary diagnosis was no other psychiatric disorder, including schizophrenia spectrum and other psychotic disorders (F20–29), mood disorders (F30–39), substance use and addictive disorders (F10–19), personality disorders (F60–69), mental retardation (F70–79), disorders of psychological development (F80–89), or neurocognitive disorders (F00–02) between 1 January 2011 and 30 June 2018 (the study period). Data of these participants collected between 1 January 2011 and 30 June 2018 were retrospectively reviewed.

The exclusion criteria were: (i) anxiety or phobic anxiety disorder or prescribed psychiatric medication between 1 January 2011 and 31 December 2012, (ii) sub-diagnosed with schizophrenia spectrum and other psychotic disorders or mood disorders between 1 January 2013 and 31 December 2014, (iii) fewer than three visits for anxiety disorders during the study period, (iv) hospitalized during the study period following the onset of anxiety disorder, and (v) death.

The prescribed psychiatric medications included the following: antidepressants (SSRIs, tricyclic antidepressants, monoamine oxidase inhibitors, SNRIs, bupropion, mirtazapine, nefazodone, tianeptine, trazodone, and agomelatine); antipsychotics (typical antipsychotics: chlorpromazine, levomepromazine, perphenazine, and haloperidol; atypical antipsychotics: ziprasidone, pimozide, clozapine, olanzapine, quetiapine, sulpiride, levosulpiride, amisulpride, risperidone, aripiprazole, and paliperidone); mood stabilizers (lithium, valproic acid, lamotrigine, topiramate, and carbamazepine); anxiolytics (diazepam, chlordiazepoxide, lorazepam, bromazepam, clobazam, alprazolam, etizolam, clotiazepam, tofisopam, and buspirone); and hypnotics and sedatives (triazolam and zolpidem).

All procedures involving human subjects/patients were approved by the Institutional Review Board of the Korea University Guro Hospital (IRB number: 2020GR0223). The need to obtain informed consent from the participants was waived owing to the observational and retrospective nature of this study.

### 2.2. Definition of Continuous Pharmacological Treatment

As HIRA does not include the specific symptoms of patients, we operationally defined the initial treatment period by investigating the termination of treatment based on prescriptions. If there was no prescription filled after 30 days from the date a certain prescription was added and the number of prescription days of that prescription, it was assumed that treatment was stopped, and the period up to the end of that prescription was defined as the initial treatment period.

Considering the low rate of continuous treatment in clinical practice, and the minimum maintenance period suggested by previous studies [19], a participant was considered to be on continuous pharmacological treatment when their initial treatment period was at least 6 months (180 days). The exact number of prescription days was counted by excluding the redundant prescription days involving multiple claims and extra days if the prescription days in the last claim during the treatment period exceeded the treatment period.

### 2.3. Definition of Relapse/Recurrence of Anxiety Disorders

We defined the relapse/recurrence of anxiety disorders by investigating the re-initiation of therapy, which included the subsequent events if other events of therapy occurred 30 days after the defined initial treatment period. Based on a retrospective cohort study investigating the relapse/recurrence of psychiatric disorders [20], the factors influencing the operational definition of re-initiation therapy, as a proxy variable for relapse/recurrence, were as follows:

The psychiatric medication prescription was resumed.

The patient was admitted for treatment of psychiatric diseases.

The patient visited the emergency department for treatment of anxiety disorders.

To analyze the variables that may affect recurrence, we defined the initial treatment period as the duration between the first prescription date of the index period and the prescription date immediately before re-initiation therapy in cases of relapse/recurrence. No relapse/recurrence was concluded when there was no re-initiation of therapy until 30 June 2018. Among the cases defined as no relapse/recurrence, 985 patients, whose last prescription date exceeded 30 June 2018, were excluded from the analysis, as they did not experience recovery without discontinuing pharmacological treatment, to focus on factors associated with relapse/recurrence. In cases of no relapse/recurrence, the initial treatment period was defined as the period between the first prescription date of the index period and the last prescription date.

### 2.4. Other Explanatory Variables

International guidelines suggest that follow-up visits for managing anxiety disorders should include monitoring symptoms and adverse reactions [18]. Therefore, we investigated the associations between the follow-up visits and the outcomes. In the absence of a specific criterion, we considered three visits for the first three months, including the initial visit for diagnosis, based on previous studies [21].

Comorbid conditions influence the prognosis of anxiety disorders. Therefore, we adopted the Charlson comorbidity index (CCIs) and calculated the score using disease codes from the ICD-10 [22]. The information on the use of drugs related to the treatment of anxiety disorders was summarized for the initial administration, final administration, and number of drug categories. As antidepressants affect discontinuation-related relapse [23], drugs were categorized into antidepressants and other drugs. Psychological treatments play an important role in the treatment of anxiety disorders, but identifying specific treatment details is difficult. The proxy variable for psychological treatment was a covariate for whether psychiatrists treated the patient, as only psychiatrists are permitted to provide psychotherapy in South Korea.

### 2.5. Statistical Method

To assess the differences in the continued antidepressant treatments, we compared the demographic and clinical characteristics, including relapse/recurrence and other explanatory variables, between those who discontinued antidepressant use early and those who received antidepressant treatment continuously, using the independent *t*-test for continuous variables and the chi-square test for categorical variables. For analyzing the associations of continuous pharmacological treatment with relapse/recurrence of anxiety disorders, we fitted a multivariable Cox proportional-hazards regression model for demographic characteristics, primary diagnosis, comorbidity, specialty of the physician, and choice of psychiatric medications. Fully adjusted hazard ratios (aHR) of the model, on which the presented results are based, were used to quantify the changes in the relative risk over time of the relapse/recurrence of anxiety disorders. Kaplan–Meier incidence plots for the main variable (the presence or absence of continuous pharmacological treatment) were constructed for visual reference. In addition to the Kaplan–Meier incidence plots, we performed a log-rank test to compare the survival distributions of the primary variable (the presence or absence of continuous pharmacological treatment) in the context of the relapse/recurrence of anxiety disorders, providing a non-parametric assessment of the differences between the two groups. For sensitivity analysis, we applied various definitions of the participants with continuous pharmacological treatment: when the participant’s initial treatment period was (a) 1 year (365 days) or more; (b) 18 months (540 days) or more; and (c) 2 years (730 days) or more. SAS 9.13 (SAS Institute, Cary, NC, USA) was used for data construction and statistical analysis, and statistical significance was determined at a significance level of 0.1% (*p* < 0.001).

## 3. Results

### 3.1. Participants

**Figure 1:**
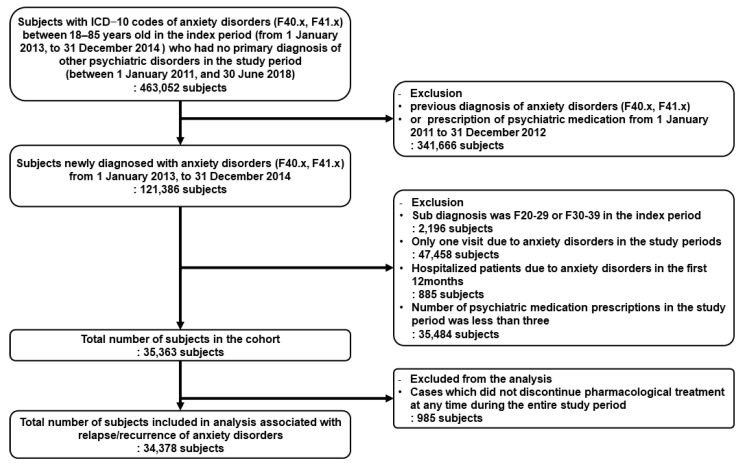
Process and criteria for selecting participants.

The number of participants in the age group of 18–85 years with anxiety disorders as the primary diagnosis in the index period and no primary diagnosis of other psychiatric disorders in the study period was 463,052; however, after considering the inclusion and exclusion criteria, 35,363 participants were selected. Additionally, after excluding cases not associated with relapse/recurrence for which pharmacological treatment was never discontinued, 34,378 patients were included in the analysis (Figure 1).

### 3.2. Comparison of Demographic and Clinical Characteristics

Among all participants, 8022 (23.33%) were categorized as the continuous pharmacological treatment group (CPG) and 26,356 (76.67%) as the early discontinued pharmacological treatment group (EPG). Table 1 shows a comparison of the demographic and clinical characteristics between the CPG and EPG. The CPG presented a significantly lower proportion of female participants (chi-square value = 26.59, *p* < 0.0001) and a higher mean age (t value = 5.08, *p* < 0.0001) than the EPG. A significantly lower proportion of CPG participants had a Charlson comorbidity index of 0 (chi-square value = 17.21, *p* = 0.0006) and a significantly greater proportion visited three times or more for the first three months (chi-square value = 2764.33, *p* < 0.0001). There was no significant difference in the proportion of primary diagnosis between F40.x and F41.x (chi-square value = 0.1624, *p* = 0.687) and the proportion of those treated by psychiatrists (chi-square value = 1.11, *p* = 0.2919).

Significant differences were observed in the initial choice of antidepressants (chi-square value = 32.70, *p* < 0.0001) and other psychiatric medications (chi-square value = 24.53, *p* < 0.0001). The CPG consisted of a higher percentage of participants with no prescription for antidepressant medication (16.13% vs. 13.61%) and a lower percentage of participants who were prescribed other psychiatric medications (16.33% vs. 17.74%) than the EPG. In the case of the last administered medication in the initial treatment period, a smaller proportion of CPG participants had no prescription for antidepressant medications (chi-square value = 47.84, *p* < 0.0001) and more had no prescription for other psychiatric medications (chi-square value = 277.19, *p* < 0.0001). During the initial treatment period, a significant number of CPG participants used more than three categories of antidepressants (chi-square value = 1875.27, *p* < 0.0001).

### 3.3. Relapse/Recurrence of Anxiety Disorders concerning Continuous Pharmacological Treatments

According to the operational definition, 59.91% of participants experienced relapse/recurrence (Supplementary Table S1). No participant continued pharmacological treatment beyond 31 June 2018. The incidence of relapse/recurrence was significantly higher in the CPG than in the EPG (chi-square value = 31.81, *p* < 0.0001). Participants with the relapse/recurrence of anxiety disorders were present in a significantly higher proportion in the following variables: sex (male, chi-square value = 10.92, *p* = 0.0009), primary diagnosis of F40.x (chi-square value = 24.53, *p* < 0.0001), specialty of physician (non-psychiatry, chi-square value = 38.01, *p* < 0.0001), and number of visits for the first 3 months (< 3, chi-square value = 24.93, *p* < 0.0001).

A significantly high number of participants with a relapse/recurrence of anxiety disorders did not use antidepressants in the initial and last medication (initial medication: chi-square value = 47.26, *p* < 0.0001; last medication: chi-square value = 269.85, *p* < 0.0001) and used one or more other psychiatric medications in the initial and last dose (initial medication: chi-square value = 48.00, *p* < 0.0001; last medication: chi-square value = 134.35, *p* < 0.0001). Those who did not experience recurrence had a higher rate of using one or more categories of antidepressants during the initial treatment period than those who did (chi-square value = 864.55, *p* < 0.0001).

### 3.4. Survival Analysis

**Figure 2:**
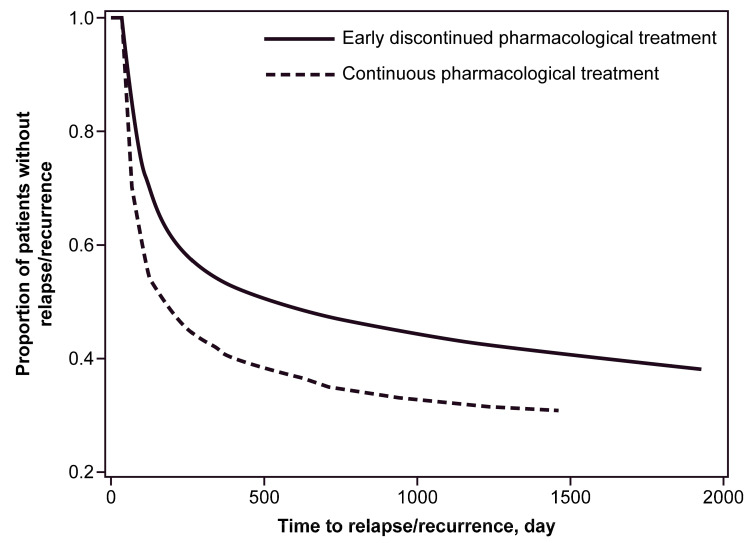
Kaplan–Meier curve for estimating time until relapse/recurrence between the continuous pharmacological treatment and early discontinued pharmacological treatment groups, compared by log-rank test.

The Kaplan–Meier curve on the difference in the risk of relapse/recurrence is illustrated in Figure 2. Participants in the CPG had a greater cumulative incidence of relapse/recurrence than those in the EPG (log-rank test: chi-square value = 244.8, *p* < 0.0001). More than 50% of patients in the CPG re-initiated treatment within 500 days from the last prescription of the initial treatment period.

Table 2 show the results of the association between relapse/recurrence and the variables, including continuous pharmacological treatment, using univariable and multivariable Cox proportional-hazards regression models with unadjusted and adjusted HRs, respectively. Significant variables in the univariate model included continuous antidepressant treatment, age, sex, primary diagnosis during initial treatment period, specialty of physicians, number of follow-up visits for the first 3 months, initial and last choice of antidepressants and other psychiatric medications, and the number of categories of antidepressants used during the initial treatment period. After adjusting for significant variables in the univariate model, the CPG had a significantly larger aHR for relapse/recurrence (1.536 (95% CI: 1.484–1.590)) than the EPG.

Patients aged 30–49 years had a slightly higher aHR or relapse/recurrence than patients aged 18–29 years. Other significant clinical variables associated with relapse/recurrence were the primary diagnosis, treatment by a psychiatrist, and clinic visits three times or more for the first three months.

The aHR was higher when antidepressants were used initially than when no antidepressants were used, and when one category of any other psychiatric medication was used at the last prescription of the initial treatment period than when no other psychiatric medication was used. Conversely, the aHR for relapse/recurrence decreased with an increase in the number of categories of antidepressants used during the initial treatment period.

The results of the sensitivity analysis to determine how the variation in the window period affects the correlation between continuous antidepressant treatment and the incidence of relapse/recurrence are presented in the Supplementary Materials (Tables S2–S4). The difference in the risk ratio of relapse/recurrence increased marginally when a longer window period was set (365 days: aHR = 1.681 (95% CI = 1.609–1.757); 540 days: aHR = 1.744 (95% CI = 1.651–1.842); 730 days: aHR = 1.782, 95% CI = 1.662–1.909). The sensitivity analysis showed that continuous antidepressant treatment was associated with an increased incidence of relapse/recurrence, irrespective of the length of the window period.

## 4. Discussion

This study suggests that recommending continuous pharmacological treatment to all patients with anxiety disorders could induce unnecessary long-term use of psychiatric medications. Additionally, the number of antidepressant categories used during the initial treatment period was significantly associated with a lower risk of relapse/recurrence. This result shows the importance of antidepressants and the necessity for active switching or addition of medication during the treatment of anxiety disorders. Furthermore, during the early phase of the treatment period, frequent visits were significantly associated with a lower rate of relapse/recurrence of the anxiety disorders.

To our knowledge, this is the first retrospective cohort study to investigate how the patterns of pharmacological treatment during the initial treatment period affect the relapse/recurrence of anxiety disorders using nationwide claim data. The study revealed that only 23.33% of newly diagnosed patients with anxiety disorders continued pharmacological treatment for more than 180 days, and 76.50% of the patients initiated treatment with a single antidepressant. These results are consistent with recent studies that emphasize the importance of sufficient antidepressant use, as the proportion of patients receiving pharmacologic treatment for anxiety disorders is low [7,24]; they are also consistent with the guideline that recommends initiating treatment with one antidepressant [25]. Caution should be exercised in interpreting the results because even a small difference could be significant in the analysis of a large sample. Therefore, we discuss only the variables that presented a markedly significant difference in the risk of relapse/recurrence (*p* < 0.0001).

### 4.1. Patterns of Pharmacological Treatment Associated with Relapse/Recurrence of Anxiety Disorders

Cox regression analysis showed a 53.6% increase in relapse/recurrence in patients who received continuous pharmacological treatment compared with those who discontinued pharmacological treatment early. These results contradict past findings that maintenance treatment with antidepressants reduces the risk of relapse in patients with anxiety disorders [26]. However, the effect size and trend did not show a difference even when the continuous pharmacological treatment window was extended to 365, 540, and 730 days in the sensitivity analysis. Moreover, setting a definitive treatment period, which we did in our study, can underestimate the risk of relapse or recurrence in claim-data-based studies [27]. In addition, there has been a recent call for evidence on the optimal duration of antidepressant treatment in anxiety disorders in the context of rapidly increasing antidepressant prescribing [28,29]. Therefore, a careful interpretation of the association of 180 days or more of antidepressant maintenance treatment with the high relapse rates found in our study based on real-world data will be important.

Our results, showing an increased risk of relapse/recurrence associated with continuous pharmacological treatment, suggest that many real-world cases may not satisfy the eligibility criteria of the RCT discontinuation studies showing the effects of the continuous use of antidepressants for preventing the relapse or recurrence of anxiety disorders [15,16]. We hypothesize that the severity of the anxiety disorder in each patient can be an important factor. Patients with higher severity are known to have longer treatment durations, poorer treatment responses, and relatively higher relapse rates [30]. The current study shows that the patient group who continued the pharmacological treatment for more than 180 days had the highest proportion of patients taking more than one category of antidepressants with psychiatric medications other than antidepressants during the initial treatment period, which could reflect the severity of their disorder [31]. This interpretation is also supported by the regression analysis results, which show that the risk of relapse was higher for those who initially took an antidepressant than for those who did not. Therefore, it can be inferred that the continuous use of antidepressants is insufficient to prevent relapse in patients with anxiety disorders and that adequate symptom control in relation to severity may be a more important factor.

Among the 26,356 patients who were treated for less than 180 days, 10,782 (40.90%) did not experience recurrence during the follow-up. The discontinuation RCT studies reported similar relapse rates in the placebo group with anxiety disorders treated for more than six months [15]. Similar to the finding of an association between continued treatment and increased risk of relapse/recurrence, our finding that a significant proportion of patients did not relapse despite the short treatment duration may suggest that the continuation of pharmacological treatment for more than 180 days for all patients with anxiety disorders might be unnecessary. Furthermore, because continuous pharmacological treatments can have adverse effects [32], these results indicate a need to develop new methods for effective relapse prevention and for screening patients who may not have relapses.

The use of antidepressants in the initial treatment period significantly reduced the risk of recurrence. Even when three or more categories of antidepressants were used, the risk of recurrence was 78% lower than when antidepressants were not used at all. This is an interesting finding, considering that the prescription of multiple antidepressants may reflect a more severe case [33]. International guidelines recommend switching or adding other antidepressants when there is no adequate treatment response [18]. Additionally, the discontinuation of pharmacological treatment in partially responsive patients with residual symptoms led to a greater risk of recurrence [34]. Therefore, these results highlight the importance of active antidepressant use during the initial treatment period.

### 4.2. Other Predictable Factors and Issues

Three or more visits to a medical institution during the first three months decreased the risk of relapse/recurrence by 20%. Short-term appointments increase patient compliance with medication [35] and help reduce symptoms [36]. Therefore, short-term visits in the early phase of treatment may have clinical significance in reducing relapse in patients with anxiety disorders.

Regarding comorbidities, the CPG group had a slightly higher proportion of higher CCIs than the EPG group. In the univariate analysis of the Cox proportional-hazards regression, the relationships between CCIs were insignificant. A comorbid diagnosis of cardiovascular or respiratory diseases and an anxiety disorder was associated with increased psychological distress and could worsen the symptoms and prognosis [37,38]. However, there are no consistent studies on the impact of the presence of comorbid diagnoses on treatment adherence or relapse and recurrence [39,40]. Conclusions concerning associations of CCIs with adherence to treatment or relapse or recurrence of an anxiety disorder cannot be drawn from our study, and further research is needed in this area.

### 4.3. Study Limitations

This study had limitations similar to other claim-data-based studies. First, we used a proxy indicator to define patients with a relapse or recurrence of anxiety disorders, which may include heterogeneous patients and is not as reliable as remission measured by clinical rating scales. Second, the effects of the residual confounding factors that were not considered cannot be completely ruled out, so it is difficult to interpret the results as a causal relationship. Third, because patients with anxiety disorders can experience stigma during treatment with psychiatric medication [41], the relapse rate seen may be lower than the actual rate. Fourth, owing to the nature of a claim-data-based study, the study population was from multiple institutions and was treated by physicians of varying specialties, leading to heterogeneity in the participants, which was beyond control. Various patient-related and supplier-related variables were also not included. Fifth, the non-random selection of the initial medication may create a selection bias in assessing factors such as adherence to treatment and rate of relapse. Sixth, consideration of the selection bias that was caused by the exclusion of the patients with more severe disease, associated with the exclusion of death cases while selecting study participants, is also necessary. Lastly, because claim records in HIRA do not show prescription dates for each drug, a detailed schedule for how each patient was dosed against time or how each patient received medication augmentation or switching was challenging to discern.

### 4.4. Clinical Implications

We show that continuous pharmacological treatment was not significantly associated with decreased relapse/recurrence after discontinuation of treatment. With a lower relapse rate in patients who discontinued pharmacological treatment early, recommending continuous pharmacological treatment to all patients with anxiety disorders can induce unnecessary use of psychiatric medications. We also suggest appropriate pharmacological treatment, with active switching and the addition of antidepressants based on the treatment response, along with frequent visits during the early phase of treatment to reduce relapse/recurrence of anxiety disorders.

## 5. Conclusions

In conclusion, our study provides important groundwork for antidepressant use in the treatment of anxiety disorders. We suggest highlighting active medication modification or addition in the treatment of anxiety disorders and attempting to avoid the unnecessary use of psychiatric medication by developing a tool for screening individuals who do not recur even after a short treatment period.

## Figures and Tables

**Table 1:** General and clinical characteristics of the participants and comparison of continuous pharmacological treatment and early discontinuation of treatment.

	Total		Early Discontinued Pharmacological Treatment (EPG)		Continuous Pharmacological Treatment (CPG)		*p*-Value ^a^
	N	(%)	N	(%)	N	(%)	
**Total**	34,378	100.00	26,356	76.67	8022	23.33	
**Age (years)**							
Mean ± standard deviation	41.57 ± 12.57		41.39 ± 12.71		42.18 ± 12.07		<0.0001
18–29	6934	20.17	5570	21.13	1364	17.00	<0.0001
30–39	8117	23.61	6148	23.33	1969	24.55	
40–49	8826	25.67	6584	24.98	2242	27.95	
50–59	7589	22.08	5814	22.06	1775	22.13	
60–69	2912	8.47	2240	8.50	672	8.38	
**Sex**							
Male	15,410	44.83	11,613	44.06	3797	47.33	<0.0001
Female	18,968	55.17	14,743	55.94	4225	52.67	
**Primary diagnosis during initial treatment period**							
F40.x	3030	8.81	2314	8.78	716	8.93	0.687
F41.x	31,348	91.19	24,042	91.22	7306	91.07	
**Charlson comorbidity index**							
0	24,839	72.25	19,170	72.73	5669	70.67	0.0006
1	5073	14.76	3859	14.64	1214	15.13	
2	3155	9.18	2362	8.96	793	9.89	
≥3	1311	3.81	965	3.66	346	4.31	
**Specialty of physician**							
Psychiatry	31,268	90.95	23,948	90.86	7320	91.25	0.2919
Non-psychiatry	3110	9.05	2408	9.14	702	8.75	
**Number of follow-up visits for the first three months**							
<3 (less than three)	11,242	32.70	10,553	40.04	689	8.59	<0.0001
3≤ (three or more)	23,136	67.30	15,803	59.96	7333	91.41	
**Initial choice of antidepressants**							
No antidepressant medications	4881	14.20	3587	13.61	1294	16.13	<0.0001
Use of one antidepressant	26,300	76.50	20,319	77.09	5981	74.56	
Combined use of antidepressants	3197	9.30	2450	9.30	747	9.31	
**Initial choice of other psychiatric medication**							
No other psychiatric medication	5985	17.41	4675	17.74	1310	16.33	<0.0001
Use of one other psychiatric medication	23,662	68.83	17,962	68.15	5700	71.05	
Combined use of other psychiatric medications	4731	13.76	3719	14.11	1012	12.62	
**Last choice of antidepressants**							
No antidepressant medications	2529	7.36	1971	7.48	558	6.96	<0.0001
Use of one antidepressant	27,292	79.39	21,074	79.96	6218	77.51	
Combined use of antidepressants	4557	13.26	3311	12.56	1246	15.53	
**Last choice of other psychiatric medication**							
No other psychiatric medication	8635	25.12	6055	22.97	2580	32.16	<0.0001
Use of one other psychiatric medication	20,790	60.47	16,424	62.32	4366	54.43	
Combined use of other psychiatric medications	4953	14.41	3877	14.71	1076	13.41	
**Number of antidepressant categories used during initial treatment period**							
0	917	2.67	910	3.45	7	0.09	<0.0001
1	21,701	63.12	17,705	67.18	3996	49.81	
2	8260	24.03	5928	22.49	2332	29.07	
≥3	3500	10.18	1813	6.88	1687	21.03	

^a^. *p*-values were obtained using chi-square tests and Student’s *t*-test.

**Table 2:** Cox proportional-hazards regression for the relapse/recurrence of anxiety disorders.

	Unadjusted HR (95% CI)	*p*-Value	Adjusted HR (95% CI)	*p*-Value
**Continuous antidepressant treatment**				
No				
Yes	1.289 (1.248–1.331)	<0.0001	1.536 (1.484–1.590)	<0.0001
**Age (years)**				
18–29				
30–39	1.139 (1.093–1.188)	<0.0001	1.131 (1.084–1.179)	<0.0001
40–49	1.117 (1.072–1.163)	<0.0001	1.096 (1.052–1.143)	<0.0001
50–59	1.07 (1.026–1.117)	0.0018	1.037 (0.993–1.083)	0.103
60–69	1.091 (1.032–1.155)	0.0023	1.057 (0.999–1.120)	0.0561
**Sex**				
Female				
Male	1.061 (1.033–1.091)	<0.0001	1.04 (1.011–1.069)	0.0056
**Primary diagnosis during initial treatment period**				
F40				
F41	0.887 (0.846–0.929)	<0.0001	0.905 (0.863–0.950)	<0.0001
**Charlson comorbidity index**				
0				
1	1.057 (1.017–1.099)	0.005		
2	1.062 (1.013–1.114)	0.0126		
≥3	1.113 (1.037–1.195)	0.003		
**Specialty of physician**				
Non-psychiatry				
Psychiatry	0.837 (0.800–0.877)	<0.0001	0.9 (0.858–0.943)	<0.0001
**Number of follow-up visits during the first three months**				
<3 (less than three)				
3≤ (three or more)	0.783 (0.762–0.806)	<0.0001	0.8 (0.776–0.825)	<0.0001
**Initial choice of antidepressants**				
No antidepressant medications				
Use of one antidepressant	0.859 (0.827–0.893)	<0.0001	1.089 (1.040–1.141)	0.0003
Combined use of antidepressants	0.788 (0.744–0.836)	<0.0001	1.215 (1.131–1.305)	<0.0001
**Initial choice of other psychiatric medication**				
No other psychiatric medication				
Use of one other psychiatric medication	1.096 (1.056–1.138)	<0.0001	1.003 (0.957–1.051)	0.8972
Combined use of other psychiatric medications	0.979 (0.930–1.029)	0.4014	1.001 (0.938–1.068)	0.9742
**Last choice of antidepressants**				
No antidepressant medications				
Use of one antidepressant	0.661 (0.629–0.693)	<0.0001	1.029 (0.962–1.101)	0.4088
Combined use of antidepressants	0.56 (0.527–0.595)	<0.0001	0.973 (0.895–1.056)	0.5087
**Last choice of other psychiatric medication**				
No other psychiatric medication				
Use of one other psychiatric medication	1.159 (1.122–1.198)	<0.0001	1.159 (1.112–1.209)	<0.0001
Combined use of other psychiatric medications	0.965 (0.921–1.011)	0.1344	1.032 (0.971–1.096)	0.3107
**Number of categories of antidepressant used during initial treatment period**				
0				
1	0.305 (0.285–0.326)	<0.0001	0.3 (0.271–0.333)	<0.0001
2	0.253 (0.235–0.271)	<0.0001	0.245 (0.220–0.272)	<0.0001
≥3	0.245 (0.226–0.266)	<0.0001	0.229 (0.204–0.256)	<0.0001

HR, Hazard ratio; CI, confidence interval.

## Data Availability

Restrictions apply to the availability of these data. Data were obtained from HIRA and are available from HIRA “https://opendata.hira.or.kr (accessed on 1 January 2021)” with the permission of HIRA.

## Supplementary Materials

The following supporting information can be downloaded at: https://www.mdpi.com/article/10.3390/life13051197/s1, Table S1: Relapse rate by general and clinical characteristics of participants; Table S2: Sensitivity analysis concerning definitions of the participants with continuous pharmacological treatment (>365 days) and Cox proportional-hazards regression for the relapse/recurrence of anxiety disorders; Table S3: Sensitivity analysis concerning definitions of the participants with continuous pharmacological treatment (>540 days) and Cox proportional-hazards regression for the relapse/recurrence of anxiety disorders; Table S4: Sensitivity analysis concerning definitions of the participants with continuous pharmacological treatment (>730 days) and Cox proportional-hazards regression for the relapse/recurrence of anxiety disorders.
