# Supplementary material for: Associations between Pharmacological Treatment Patterns during the Initial Treatment Period and the Relapse or Recurrence of Anxiety Disorders: A Nationwide Retrospective Cohort Study
Source: Life (Basel). 2023 May 17;13(5):1197. doi: 10.3390/life13051197 (PMC10220856; doi:10.3390/life13051197)
Supplement: Supplementary file 1 [file life-13-01197-s001.zip › life-2315901-supplementary.pdf]

Supplementary Materials

Supplementary Table S1. Relapse rate by general and clinical characteristics of participants

	No relapse/recurrence		Relapse/recurrence		*P-value* [a]
	N	(%)	N	(%)	
**Total**	13,781	40.09	20,597	59.91	
**Continuous pharmacological treatment**					
No	10,782	78.24	15,574	75.61	<0.0001
Yes	2,999	21.76	5,023	24.39	
**Age (years)**					
Mean ± standard deviation	41.40±12.79		41.69±12.42		0.0421
18–29	2,972	21.57	3,962	19.24	<0.0001
30–39	3,109	22.56	5,008	24.31	
40–49	3,470	25.18	5,356	26.00	
50–59	3,073	22.30	4,516	21.93	
60–69	1,157	8.40	1,755	8.52	
**Sex**					
Male	6,028	43.74	9,382	45.55	0.0009
Female	7,753	56.26	11,215	54.45	
**Primary diagnosis during initial treatment period**					
F40	1,086	7.88	1,944	9.44	<0.0001
F41	12,695	92.12	18,653	90.56	
**Charlson comorbidity index**[9]					
0	10,102	73.30	14,737	71.55	0.0052
1	1,956	14.19	3,117	15.13	

2	1,221	8.86	1,934	9.39	
≥3	502	3.64	809	3.93	
**Specialty of physician**					
Psychiatry	12,695	92.12	18,573	90.17	<0.0001
Non-psychiatry	1,086	7.88	2,024	9.83	
**Number of follow-up visits for the first 3 months**					
<3 (less than three)	3,471	25.19	7,771	37.73	<0.0001
3≤ (three or more)	10,310	74.81	12,826	62.27	
**Initial choice of antidepressants**					
No antidepressant medications	1,777	12.89	3,104	15.07	<0.0001
Use of one antidepressant	10,601	76.92	15,699	76.22	
Combined use of antidepressants	1,403	10.18	1,794	8.71	
**Initial choice of other psychiatric medication**					
No other psychiatric medication	2,526	18.33	3,459	16.79	<0.0001
Use of one other psychiatric medication	9,199	66.75	14,463	70.22	
Combined use of other psychiatric medication	2,056	14.92	2,675	12.99	
**Last antidepressant prescription of initial treatment period**					
No antidepressants medications	683	4.96	1,846	8.96	<0.0001
Use of one antidepressant	10,958	79.52	16,334	79.30	
Combined use of antidepressants	2,140	15.53	2,417	11.73	
**Last other psychiatric medication prescription of initial treatment period**					
No other psychiatric medication	3,713	26.94	4,922	23.90	<0.0001
Use of one other psychiatric medication	7,831	56.82	12,959	62.92	

Combined use of other psychiatric medication	2,237	16.23	2,716	13.19	
**Number of used categories of antidepressant during initial treatment period**					
0	-	0.00	917	4.45	<0.0001
1	8,294	60.18	13,407	65.09	
2	3,785	27.47	4,475	21.73	
≥3	1,702	12.35	1,798	8.73	

[a.] p-value were obtained using chi-square tests and Student's t-tests.

Supplementary Table S2. Sensitivity analysis concerning definitions of the participant with continuous pharmacological treatment (>365 days).

Cox proportional hazard regression for the relapse/recurrence of anxiety disorders

	**Unadjusted HR (95% CI)**	**p-value**	**Adjusted HR (95% CI)**	**p-value**
**Continuous antidepressant treatment**				
No				
Yes	1.436 (1.377-1.497)	<0.0001	1.681 (1.609-1.757)	<0.0001
**Age (years)**				
18–29				
30–39	1.139 (1.093–1.188)	<0.0001	1.129 (1.082–1.177)	<0.0001
40–49	1.117 (1.072–1.163)	<0.0001	1.098 (1.054–1.145)	<0.0001
50–59	1.07 (1.026–1.117)	0.0018	1.038 (0.994–1.085)	0.0917
60–69	1.091 (1.032–1.155)	0.0023	1.058 (0.999–1.121)	0.0533
**Sex**				
Female				
Male	1.061 (1.033–1.091)	<0.0001	1.044 (1.015–1.073)	0.0024

**Primary diagnosis during initial treatment period**				
F40				
F41	0.887 (0.846–0.929)	<0.0001	0.902 (0.860–0.946)	<0.0001
**Charlson comorbidity index[9]**				
0				
1	1.057 (1.017–1.099)	0.005		
2	1.062 (1.013–1.114)	0.0126		
≥3	1.113 (1.037–1.195)	0.003		
**Specialty of physician**				
Non-psychiatry				
Psychiatry	0.837 (0.800–0.877)	<0.0001	0.901 (0.859–0.944)	<0.0001
**Number of follow-up visits during the first three months**				
<3 (less than three)				
3≤ (three or more)	0.783 (0.762–0.806)	<0.0001	0.8 (0.776–0.825)	<0.0001
**Initial choice of antidepressants**				

No antidepressant medications				
Use of one antidepressant	0.859 (0.827–0.893)	<0.0001	1.085 (1.036–1.136)	0.0003
Combined use of antidepressants	0.788 (0.744–0.836)	<0.0001	1.209 (1.126–1.299)	<0.0001
**Initial choice of other psychiatric medication**				
No other psychiatric medication				
Use of one other psychiatric medication	1.096 (1.056–1.138)	<0.0001	1.003 (0.957–1.051)	0.8972
Combined use of other psychiatric medication	0.979 (0.930–1.029)	0.4014	1.002 (0.939–1.070)	0.9742
**Last choice of antidepressants**				
No antidepressant medications				
Use of one antidepressant	0.661 (0.629–0.693)	<0.0001	1.029 (0.962–1.101)	0.4088
Combined use of antidepressants	0.56 (0.527–0.595)	<0.0001	0.973 (0.895–1.056)	0.5087
**Last choice of other psychiatric medication**				
No other psychiatric medication				
Use of one other psychiatric medication	1.159 (1.122–1.198)	<0.0001	1.146 (1.099–1.195)	<0.0001

Combined use of other psychiatric medication	0.965 (0.921–1.011)	0.1344	1.013 (0.954–1.076)	0.3107
**Number of used categories of antidepressant during initial treatment period**				
0				
1	0.305 (0.285–0.326)	<0.0001	0.309 (0.279–0.342)	<0.0001
2	0.253 (0.235–0.271)	<0.0001	0.253 (0.228–0.282)	<0.0001
≥3	0.245 (0.226–0.266)	<0.0001	0.238 (0.212–0.266)	<0.0001

Supplementary Table S3. Sensitivity analysis concerning definitions of the participant with continuous pharmacological treatment (>540 days). Cox proportional hazard regression for the relapse/recurrence of anxiety disorders

	**Unadjusted HR (95% CI)**	**p-value**	**Adjusted HR (95% CI)**	**p-value**
**Continuous antidepressant treatment**				
No				
Yes	1.500 (1.423-1.581)	<0.0001	1.744 (1.651-1.842)	<0.0001
**Age (years)**				
18–29				
30–39	1.139 (1.093–1.188)	<0.0001	1.134 (1.087–1.182)	<0.0001
40–49	1.117 (1.072–1.163)	<0.0001	1.105 (1.060–1.152)	<0.0001
50–59	1.07 (1.026–1.117)	0.0018	1.043 (0.998–1.090)	0.0587
60–69	1.091 (1.032–1.155)	0.0023	1.064 (1.005–1.127)	0.0340
**Sex**				
Female				
Male	1.061 (1.033–1.091)	<0.0001	1.045 (1.016–1.074)	0.0019

**Primary diagnosis during initial treatment period**				
F40				
F41	0.887 (0.846–0.929)	<0.0001	0.898 (0.856–0.942)	<0.0001
**Charlson comorbidity index[9]**				
0				
1	1.057 (1.017–1.099)	0.005		
2	1.062 (1.013–1.114)	0.0126		
≥3	1.113 (1.037–1.195)	0.003		
**Specialty of physician**				
Non-psychiatry				
Psychiatry	0.837 (0.800–0.877)	<0.0001	0.898 (0.857–0.941)	<0.0001
**Number of follow-up visits during the first three months**				
<3 (less than three)				
3≤ (three or more)	0.783 (0.762–0.806)	<0.0001	0.849 (0.824–0.875)	<0.0001
**Initial choice of antidepressants**				

No antidepressant medications				
Use of one antidepressant	0.859 (0.827–0.893)	<0.0001	1.079 (1.030–1.130)	0.0013
Combined use of antidepressants	0.788 (0.744–0.836)	<0.0001	1.195 (1.112–1.284)	<0.0001
**Initial choice of other psychiatric medication**				
No other psychiatric medication				
Use of one other psychiatric medication	1.096 (1.056–1.138)	<0.0001	1.014 (0.967–1.063)	0.5650
Combined use of other psychiatric medication	0.979 (0.930–1.029)	0.4014	1.010 (0.946–1.078)	0.7696
**Last choice of antidepressants**				
No antidepressant medications				
Use of one antidepressant	0.661 (0.629–0.693)	<0.0001	1.009 (0.943–1.079)	0.7984
Combined use of antidepressants	0.56 (0.527–0.595)	<0.0001	0.947 (0.872–1.028)	0.1951
**Last choice of other psychiatric medication**				
No other psychiatric medication				
Use of one other psychiatric medication	1.159 (1.122–1.198)	<0.0001	1.124 (1.078–1.172)	<0.0001

Combined use of other psychiatric medication	0.965 (0.921–1.011)	0.1344	0.996 (0.937–1.058)	0.8873
**Number of used categories of antidepressant during initial treatment period**				
0				
1	0.305 (0.285–0.326)	<0.0001	0.315 (0.284–0.349)	<0.0001
2	0.253 (0.235–0.271)	<0.0001	0.261 (0.234–0.290)	<0.0001
≥3	0.245 (0.226–0.266)	<0.0001	0.248 (0.221–0.277)	<0.0001

Supplementary Table S4. Sensitivity analysis concerning definitions of the participant with continuous pharmacological treatment (>730 days).

Cox proportional hazard regression for the relapse/recurrence of anxiety disorders

	**Unadjusted HR (95% CI)**	**p-value**	**Adjusted HR (95% CI)**	**p-value**
**Continuous antidepressant treatment**				
No				
Yes	1.533 (1.433-1.640)	<0.0001	1.782 (1.662-1.909)	<0.0001
**Age (years)**				
18–29				
30–39	1.139 (1.093–1.188)	<0.0001	1.139 (1.092–1.188)	<0.0001
40–49	1.117 (1.072–1.163)	<0.0001	1.110 (1.065–1.157)	<0.0001
50–59	1.07 (1.026–1.117)	0.0018	1.049 (1.004–1.096)	0.0314
60–69	1.091 (1.032–1.155)	0.0023	1.067 (1.008–1.130)	0.0264
**Sex**				
Female				
Male	1.061 (1.033–1.091)	<0.0001	1.047 (1.019–1.077)	0.0010

**Primary diagnosis during initial treatment period**				
F40				
F41	0.887 (0.846–0.929)	<0.0001	0.897 (0.855–0.941)	<0.0001
**Charlson comorbidity index**[9]				
0				
1	1.057 (1.017–1.099)	0.005		
2	1.062 (1.013–1.114)	0.0126		
≥3	1.113 (1.037–1.195)	0.003		
**Specialty of physician**				
Non-psychiatry				
Psychiatry	0.837 (0.800–0.877)	<0.0001	0.898 (0.857–0.941)	<0.0001
**Number of follow-up visits during the first three months**				
<3 (less than three)				
3≤ (three or more)	0.783 (0.762–0.806)	<0.0001	0.858 (0.833–0.885)	<0.0001
**Initial choice of antidepressants**				

No antidepressant medications				
Use of one antidepressant	0.859 (0.827–0.893)	<0.0001	1.078 (1.029–1.129)	0.0015
Combined use of antidepressants	0.788 (0.744–0.836)	<0.0001	1.187 (1.105–1.275)	<0.0001
**Initial choice of other psychiatric medication**				
No other psychiatric medication				
Use of one other psychiatric medication	1.096 (1.056–1.138)	<0.0001	1.021 (0.974–1.070)	0.3952
Combined use of other psychiatric medication	0.979 (0.930–1.029)	0.4014	1.015 (0.951–1.083)	0.6611
**Last choice of antidepressants**				
No antidepressant medications				
Use of one antidepressant	0.661 (0.629–0.693)	<0.0001	1.000 (0.935–1.070)	0.9971
Combined use of antidepressants	0.56 (0.527–0.595)	<0.0001	0.938 (0.864–1.019)	0.1314
**Last choice of other psychiatric medication**				
No other psychiatric medication				
Use of one other psychiatric medication	1.159 (1.122–1.198)	<0.0001	1.112 (1.067–1.160)	<0.0001

Combined use of other psychiatric medication	0.965 (0.921–1.011)	0.1344	0.986 (0.928–1.047)	0.6455
**Number of used categories of antidepressant during initial treatment period**				
0				
1	0.305 (0.285–0.326)	<0.0001	0.319 (0.288–0.354)	<0.0001
2	0.253 (0.235–0.271)	<0.0001	0.266 (0.239–0.296)	<0.0001
≥3	0.245 (0.226–0.266)	<0.0001	0.256 (0.229–0.287)	<0.0001
